# TRPV1 Agonist Capsaicin Enhances Oxidative-Stress Resistance and Regeneration in Dorsal Root Ganglia and Schwann Cells

**DOI:** 10.3390/cells15131142

**Published:** 2026-06-24

**Authors:** Baffour Kyei Sarpong, Niklas Rilke, Lea Joswig, Finn Specht, Mona Shaygan Tabar, Alina Blusch, Anna Meichsner, Pia Renk, Xiomara Pedreiturria, Thomas Grüter, Rafael Klimas, Konstanze F. Winklhofer, Ralf Gold, Melissa Sgodzai, Kalliopi Pitarokoili

**Affiliations:** 1 Department of Neurology, St. Josef-Hospital, Ruhr University Bochum, Gudrunstr. 56, 44791 Bochum, Germany; 2 Brain Disease Biomarker Unit, Wallenberg Neuroscience Center, Department of Experimental Medical Science, Medical Faculty, Lund University, 223 62 Lund, Sweden; 3 Department of Molecular Cell Biology, Institute of Biochemistry and Pathobiochemistry, Ruhr University Bochum, 44801 Bochum, Germany; 4 Department of Neurology and Stroke Unit, Klinikum Lippstadt, 59555 Lippstadt, Germany

**Keywords:** capsaicin, dorsal root ganglia, Schwann cells, chronic inflammatory demyelinating polyneuropathy, regeneration, oxidative stress

## Abstract

**Highlights:**

**What are the main findings?**

- Capsaicin promotes TRPV1-mediated neuroprotection and neuroregeneration of dorsal root ganglia neurons through activation of catalase (*Cat*) and *Nrf2*, *Ho-1*, *Nqo1* antioxidant cascade and regeneration associated molecules *Gap43* and *Atf3*.
- Capsaicin modulates Schwann cell mitochondrial bioenergetics and repair and myelination phenotypes oxidative stress conditions.

**What are the implications of the main findings?**

- TRPV1-mediated capsaicin signaling serves as a mechanistic link between oxidative stress adaptation and peripheral nerve regeneration.
- Capsaicin-mediated modulation of neuronal oxidative stress response and Schwann cell bioenergetics and plasticity highlights its therapeutic potential for inflammatory and degenerative neuropathies characterized by oxidative injury.

**Abstract:**

Neurodegeneration and oxidative stress are central drivers of immune-mediated neuropathies. Capsaicin, the active ingredient in chili pepper and a direct agonist of the transient receptor potential vanilloid (TRPV1) channel, is used clinically to treat neuropathic pain. We previously demonstrated immunomodulatory and antioxidative effects of capsaicin in experimental autoimmune neuritis in vivo and Schwann cells (SC) in vitro. However, the molecular mechanisms underlying the maintenance of axonal integrity in dorsal root ganglion (DRG) and SC homeostasis remain unclear. In this study, we described the effects of capsaicin on DRG and SC in vitro under both naïve and S-Nitroso-N-acetyl-DL-penicillamine (SNAP)-induced oxidative stress conditions. Capsaicin induced an upregulation of the antioxidative cascade involving *Nrf2*, *Ho-1*, and *Nqo1* in naïve DRG neurons and restored axonal growth under preventive and therapeutic settings. Preventive treatment enhanced catalase expression, whereas treatment increased regeneration-associated *Gap43* and *Atf3*. Inhibition of TRPV1 with capsazepine partly attenuated the protective effect of axonal outgrowth, indicating TRPV1-mediated neuroprotection. In SC, capsaicin increased mitochondrial ATP production and spare respiratory capacity, inducing a transient *Nrf2*-dependent antioxidant response. Capsaicin suppressed expression of myelination markers under basal conditions but promoted expression of myelination- and repair-associated markers under oxidative stress. The findings support capsaicin as a regulator of neuronal and Schwann cell oxidative stress adaptation.

## 1. Introduction

Neurodegeneration is the main driver of chronic disability for disorders of the peripheral nervous system (PNS) [1,2,3]. The mechanisms of neurodegeneration include primary or secondary degeneration, such as in the context of inflammation. Oxidative stress is shared across all PNS diseases and is associated with increased reactive oxygen species and decreased endogenous antioxidant levels [4,5,6,7]. Although a variety of anti-inflammatory therapies are available to regulate inflammation in the PNS, little is known about treatment options targeting oxidative stress or directly promoting regeneration [8,9,10].

The impact of environmental and nutritional factors on immune-mediated diseases of the PNS has gained significant attention in recent years [11,12]. Based on epidemiological studies, there is a trend of a higher incidence of acute inflammatory demyelinating polyneuropathy (Guillain-Barré Syndrome, GBS) in Europe and North America in comparison to China and Brazil [13,14,15], countries with high consumption of capsaicin in spicy food. Capsaicin is the active component of chili pepper, with a mode of action mediated by the transient receptor potential channel vanilloid subfamily member 1 (TRPV1), a non-selective calcium-permeable cation channel, which is expressed both in the nervous system and the immune system [16,17,18,19,20].

Our group has shown profound effects of capsaicin in the PNS, which encompasses an immunomodulatory effect of a capsaicin-enriched diet in the rat model of immune-mediated neuropathies (experimental autoimmune neuritis, EAN) with a decrease in proinflammatory tumor necrosis factor-a (TNF-α) and an increase in TRPV1 expression in the sciatic nerves [21], as well as direct anti-inflammatory and antioxidative effects in a Schwann cell (SC) culture [22].

TRPV1 expression on dorsal root ganglia (DRG) has been extensively investigated in the context of nociceptive pain. A high-concentration capsaicin transdermal patch is used for the treatment of nociceptive pain in a variety of polyneuropathies [23,24,25]. An increase in intradermal nerve fiber innervation was detected three months after the application of the transdermal patch in patients with painful diabetic neuropathy, which provides evidence for secondary regenerative effects of capsaicin after its initial anti-nociceptive actions [26].

Here, we focus on antioxidative and regenerative mechanisms of capsaicin on DRG and SC in vitro and describe a TRPV1-mediated mode of action.

## 2. Materials and Methods

### 2.1. Animal Experiments

We carried out all animal experiments in accordance with the European Communities Council Directive of 22 September 2010 (2010/63/EEC) for the care of laboratory animals and with the consent of the local government committee for nature, environment, and consumer protection.

We housed male Sprague–Dawley rats (3 to 4 weeks old, Charles River, RRID: RGD_1566457) in a temperature and humidity-controlled vivarium with a constant 12 h-light-dark cycle with free access to food and water.

### 2.2. Drugs

Capsaicin (Alps Pharmaceutical, Hida, Japan, 93.1% pure powder) was dissolved in dimethyl sulfoxide (DMSO) as instructed by the supplier and used in concentrations of 0.1, 1, 10, 100, and 1000 µM; the DMSO concentration was maintained at 0.1% across all conditions. Capsazepine (MedChemExpress, Monmouth Junction, NJ 08852, USA, 99.05% pure powder), a TRPV1 antagonist, was used after initial titration to exclude toxic effects at a concentration of 10 µM diluted in DMSO. To induce oxidative stress in vitro, the nitric oxide donor S-Nitroso-N-acetyl-DL-penicillamine (SNAP) was used at a concentration of 2 mM in Schwann cell mRNA expression analysis according to proper cell-death titration. In the mitochondrial respiration analysis, fewer Schwann cells were used, and subsequently SNAP was used at a concentration of 500 µM. The same concentration of SNAP was used for the DRG experiments [8].

### 2.3. Isolation, Outgrowth, and Staining of DRG

We harvested DRGs and cultured them in DRG growth medium as described previously [8]. Briefly, we incubated single DRG explants at 37 °C and 5% CO_2_ in neurobasal medium (Life Technologies, Carlsbad, CA, USA) supplemented with 2% B27 (Life Technologies), 2% normal horse serum (Thermo Fischer Scientific, Waltham, MA, USA), 1% l-glutamine (Thermo Fischer Scientific), 50 µg/mL Gentamicin (Thermo Fischer Scientific), and 20 ng/mL neuronal growth factor (Merck, Darmstadt, Germany). For immunohistochemistry, we fixed the DRGs with 4% paraformaldehyde (PFA) and blocked them with blocking solution containing 10% goat serum and 0.05% Triton X-100, and incubated them overnight with βIII tubulin primary antibody (1:4000, T2200, RRID: AB_262133, Sigma-Aldrich, St. Louis, MO, USA) followed by the appropriate secondary antibodies. We assessed explant outgrowth by measuring the lengths of the 10 longest neurites with the NeuronJ^®^ plugin (http://www.imagescience.org/meijering/software/neuronj/, RRID:SCR_002074, accessed on 29 June 2024). Although DRG explants contain large numbers of neurons, measuring the ten longest neurites per explant provides a standardized and commonly used regenerative outgrowth that determines treatment-dependent differences and minimizes neuron-selection bias [8,21,27,28,29].

### 2.4. Isolation, Purification, and Cultivation of Schwann Cells

We prepared the SC as described in [22,30]. The rats’ sciatic nerves were initially harvested into sterile PBS and transferred to Leibovitz’s medium enriched with 50 μg/mL Gentamicin (Thermo Fisher Scientific, Waltham, MA, USA). The nerves were cleaned of blood, foreign materials, and epineurium and carefully teased apart. Then we digested the sciatic nerves overnight with Dispase II (Sigma Aldrich, Saint Louis, MO, USA) and Collagenase I (Sigma Aldrich). The resulting cell suspension was then incubated in a solution containing 2 µM forskolin (Sigma-Aldrich) and 10 nM neuregulin (PeproTech, Rocky Hill, NJ, USA) to induce differentiation. Fibroblasts were separated from the cells through magnetic cell selection to achieve a Schwann cell purity of above 95%.

### 2.5. Capsaicin-Mediated Neuroprotection Against Oxidative Stress in DRG

After isolation, the DRGs were kept for 24 h in a growth medium containing 10 μM capsaicin or DMSO as a control, then treated with 500 µM SNAP in a fresh medium for another 24 h to induce oxidative stress. Immunohistochemistry and neurite outgrowth were carried out as described above. To assess if neuroprotection is mediated via the TRPV1 channel, we used receptor antagonist capsazepine at a concentration of 10 µM in the same experimental setup, with application of capsazepine for 30 min before treatment with capsaicin. Preventive treatment design, as an assessment of neuroprotection, asks whether capsaicin can preserve neuronal integrity and viability and limit oxidative-stress injury before or during the existence of the injury.

### 2.6. Capsaicin-Mediated Neuroregeneration After Oxidative Stress in DRG

The DRGs were cultivated in a growth medium. After 24 h, the medium was replaced by 500 µM SNAP in a fresh DRG growth medium and incubated for another 24 h to induce oxidative stress. Then they were treated with capsaicin by replacing the medium with DRG growth medium containing 10 μM capsaicin or DMSO as a control and cultured for an additional 24 h. The immunohistochemistry and neurite outgrowth were assessed as described above. Therapeutic treatment design, as an assessment of neuroregeneration, asks whether capsaicin can support the recovery of neurites following oxidative stress injury.

### 2.7. Mitochondrial Respiration Analysis in Schwann Cells

Real-time mitochondrial activity in SC was analyzed using the Seahorse XF Pro analyzer with the Mitostress test and the Glycolytic stress test. Mitochondrial and glycolytic parameters, including spare respiratory capacity, ATP-linked respiration, glycolytic flux, and non-glycolytic acidification, were quantified as described by the manufacturer. Schwann cells were seeded into Seahorse XF Pro M 96-well microplates at a density of 15,000 cells/well, with the peripheral wells reserved for background normalization, and maintained for 5 days before analysis. The sensor cartridge was calibrated 24 h before measurements by incubation with 200 µL of Seahorse XF calibrant per well at 37 °C in a CO_2_-free environment. One hour before data acquisition, the culture medium was replaced with Seahorse XF DMEM assay medium (pH 7.4) supplemented with 2 mM glutamine. For mitochondrial stress experiments, the medium was also enriched with 10 mM glucose and 1 mM pyruvate. Cells were then maintained under CO_2_-free conditions until the start of the assay. Test reagents were prepared at tenfold working concentrations. Data analysis and acquisition were conducted using Wave Pro software version 10.2.1 following the manufacturer’s guidelines.

### 2.8. Effect of Capsaicin on DRG and Schwann Cells on mRNA Level

We performed real-time PCR in DRG after treatment with capsaicin for 8 h and 24 h, respectively, to analyze whether capsaicin regulates *Trpv1*, cellular antioxidant defense proteins, and regeneration-associated neuronal markers. DRG and Schwann cells were also analyzed with or without SNAP application before treatment with capsaicin for 24 h.

The experimental procedure was described previously [8]. Briefly, total mRNA was isolated using the RNeasy Mini Kit (Qiagen, Naamloze, The Netherlands) and stored at −80 °C until further processing. cDNA was synthesized according to the manufacturer’s protocol using the Reverse Transcription System (Promega, Walldorf, Germany). Sequence-specific sense and antisense primers were then designed, as listed in the table below (Table 1), and mRNA expression levels were quantified by qRT-PCR following the manufacturer’s instructions (Thermo Fisher Scientific).

Quantitative mRNA levels were estimated by normalizing target-gene Ct values to the geometric mean of reference-gene expression of *Actb* and *Gapdh*. All the measurements were performed in triplicate, and the average Ct values were used in subsequent statistical analyses.

### 2.9. Statistics

We used GraphPad Prism 10 software (GraphPad Software Inc., 11.0.1.90 http://www.graphpad.com/, RRID:SCR_002798) for statistical analysis, the Shapiro–Wilk test for normality analysis of data, the Student’s *t* test to test differences between pairs of groups when data were normally distributed, and the Mann–Whitney test for non-normal distributions. Differences between three or more groups were tested by ANOVA if normally distributed, and the Kruskal–Wallis test if not normally distributed. Data is provided as mean ± SD.

## 3. Results

### 3.1. Capsaicin Increases DRG Resistance to Oxidative Stress by Catalase Expression

**Figure 1:**
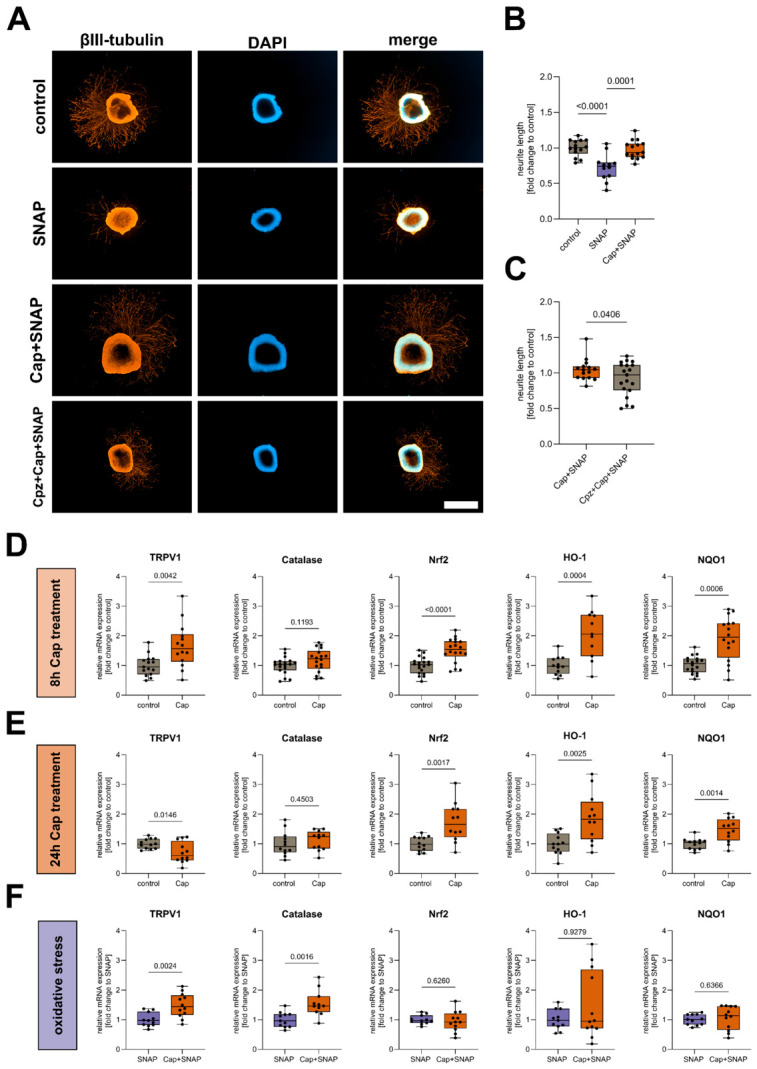
Capsaicin confers neuroprotection to DRG neurons by enhancing neurite outgrowth through activation of antioxidative pathways that increase oxidative stress resistance. (**A**) Representative images of neurite outgrowth under naïve conditions, SNAP-induced oxidative stress, capsaicin-pretreated DRG under SNAP-induced oxidative stress, and capsazepine-pretreated DRG before capsaicin and SNAP treatment. Quantification of neurite outgrowth showing increased axonal DRG outgrowth in capsaicin-pretreated DRG after SNAP application (**B**) and the abolished neuroprotective effects of capsaicin by prior capsazepine treatment (**C**). Relative mRNA expression of *Trpv1*, catalase (*Cat*), *Nrf2*, *Ho-1*, and *Nqo1* in DRG after 8 h and 24 h capsaicin treatment under naïve conditions (**D**,**E**, respectively). Relative mRNA expression of *Trpv1*, catalase (*Cat*), *Nrf2*, *Ho-1*, and *Nqo1* treated with capsaicin under SNAP-induced oxidative stress (**F**). Statistical significance was assessed by one-way ANOVA (**B**) and Student’s *T*-test (**C**–**F**). Data is provided as mean ± SD.

DRG explants have been used in several studies as a standardized method for axonal growth capacity for more than a decade [31]. Under naïve conditions, DRG explant neurons exhibited an average axonal outgrowth of 771.43 ± 143.05 μm after 24 h. (Figure 1A). The induction of oxidative stress with S-Nitroso-N-acetyl-DL-penicillamine (SNAP) led to a significant reduction in axonal outgrowth by approximately 25.2% (SNAP, *p* < 0.0001, *n* = 12, Figure 1B). A 24 h pre-treatment with capsaicin increased neurite outgrowth under oxidative stress conditions, suggesting a potential neuroprotective effect. (*p* = 0.0001, *n* = 15, Figure 1B).

To assess whether these antioxidative effects rely on TRPV1 activation by capsaicin, we used the receptor antagonist capsazepine. Capsazepine pretreatment partially attenuated the protective effect of capsaicin (*p* = 0.0406, *n* = 13, Figure 1C).

Given the enhanced oxidative stress tolerance observed in capsaicin-treated DRG, we performed quantitative mRNA expression analysis to examine the expression of known antioxidants like Catalase (*Cat*), nuclear factor Erythroid-2 related factor 2 (*Nrf2*), NAD(P)H quinone dehydrogenase 1 (*Nqo1*), heme oxygenase 1 (*Ho-1*), and the *Trpv1* receptor.

Already 8 h after treatment, significant upregulation of *Trpv1* (*p* = 0.0042, *n* = 6, Figure 1D), *Nrf2* (*p* < 0.0001, *n* = 6, Figure 1D), *Ho-1* (*p* = 0.0004, *n* = 6, Figure 1D), and *Nqo1* (*p* < 0.0006, *n* = 6, Figure 1D) was detected. The altered regulation of antioxidative proteins *Nrf2*, *Ho-1*, and *Nqo1* also continued 24 h after treatment with capsaicin, but in a reduced level of significance compared to control (*Nrf2*: *p* = 0.0017, *n* = 6, Figure 1E; *Ho-1*: *p* = 0.0025, *n* = 6, Figure 1E; *Nqo1*: *p* < 0.0014, *n* = 6, Figure 1E). In contrast to this, the expression of *Trpv1* was significantly downregulated 24 h after treatment with capsaicin (*p* < 0.0146, *n* = 6, Figure 1E). The expression of catalase (*Cat*) remained unaffected at both timepoints (Figure 1D,E). Under oxidative stress conditions, both the *Trpv1* receptor (*p* = 0.002, *n* = 12, Figure 1F) and catalase (*Cat*) (*p* = 0.002, *n* = 11, Figure 1F) were significantly upregulated after capsaicin treatment. We did not detect further significant changes in the relative mRNA expression of *Nfr2*, *Ho-1*, and *Nqo1* after capsaicin treatment (Figure 1F).

### 3.2. Capsaicin Induces Neuroregeneration via Growth-Associated Protein 43 (GAP43) and Activation of Transcription Factor 3 (ATF3)

**Figure 2:**
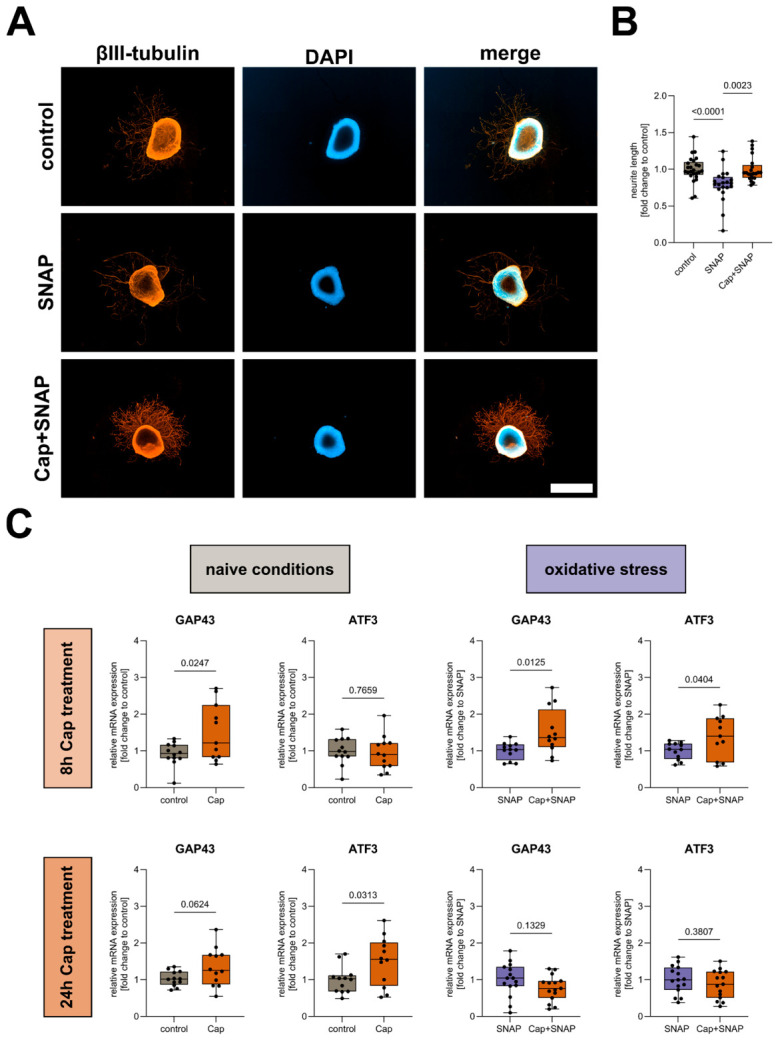
Capsaicin promotes neuroregenerative effects in DRG neurons following oxidative stress. (**A**) Representative images of neurite outgrowth from DRG explants treated with SNAP and followed by capsaicin. (**B**) Quantification of neurite outgrowth showing an enhanced axonal regeneration by capsaicin after SNAP exposure. (**C**) Relative mRNA expression of regenerative markers *Gap43* and *Atf3* under naïve and oxidative stress conditions at 8 h and 24 h timepoints. Statistical significance was assessed by the Kruskal–Wallis test with multiple comparisons (**B**) and Student’s *T*-test (**C**). Data is provided as mean ± SD.

Application of capsaicin for 24 h after oxidative stress induction with SNAP led to increased axonal outgrowth, indicating the promotion of regenerative processes. (*p* = 0.0023, *n* = 15, Figure 2A,B). Consequently, mRNA expression analysis was conducted to investigate regeneration-associated proteins like growth-associated protein 43 (*Gap43*) and activating transcription factor 3 (*Atf3*). Under naïve conditions, *Gap43* shows an early response to the capsaicin treatment, since it is significantly upregulated after 8 h (*p* = 0.0247, *n* = 6, Figure 2C), whereas *Atf3* is not alternatively expressed after 8 h, but significantly increased after 24 h upon capsaicin treatment (*p* = 0.0313, *n* = 6, Figure 2C).

The examination of both targets under oxidative stress conditions induced via SNAP shows significant upregulation of both *Gap43* (*p* = 0.0125, *n* = 6, Figure 2C) and *Atf3* (*p* = 0.0404, *n* = 6, Figure 2C) after 8 h, but not significant alteration after 24 h.

### 3.3. Capsaicin Leads to the Induction of an Anti-Oxidative Response in Schwann Cells via Changes in Mitochondrial Respiration

Previous work from our group demonstrated immunomodulatory and antioxidative effects of capsaicin in Schwann cells [22]. Complementary to this work and our pathway analysis in DRG, we examined the regulation of antioxidative actions in Schwann cells after capsaicin treatment.

**Figure 3:**
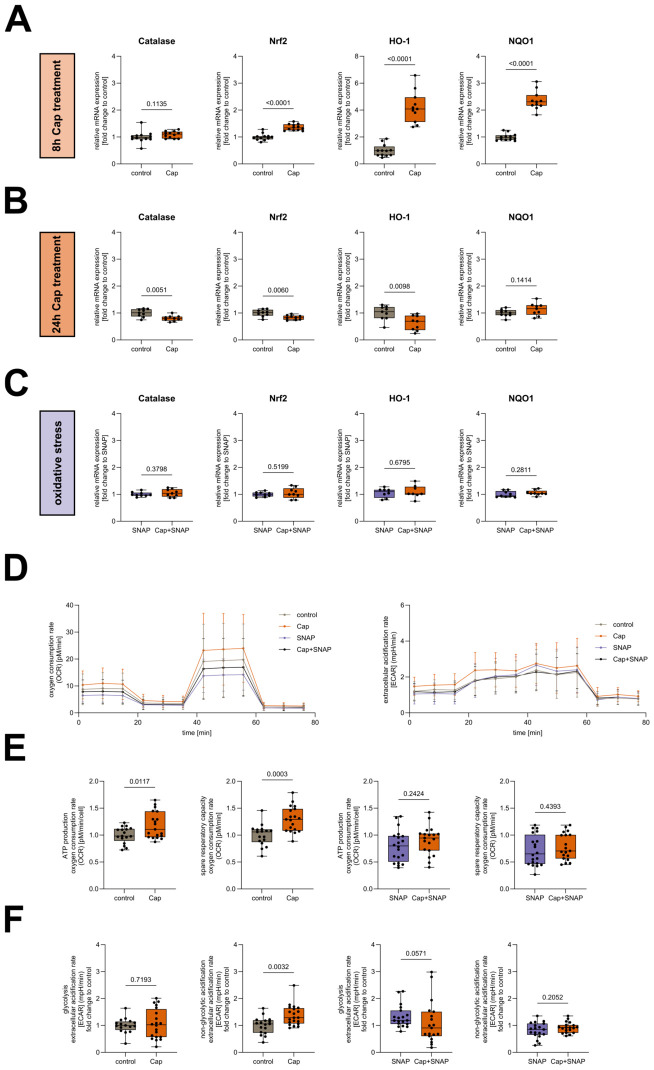
Capsaicin modulates oxidative-stress signaling and mitochondrial bioenergetics under naïve and oxidative-stress conditions. (**A**,**B**) Relative mRNA expression of catalase, *Nrf2*, *Ho-1*, and *Nqo1* in SC following 8 h and 24 h treatment with capsaicin under naïve conditions, respectively. (**C**) Relative mRNA expression of antioxidative markers catalase, *Nrf2*, *Ho-1*, and *Nqo1* in SC treated with capsaicin under oxidative stress. (**D**) Representative Seahorse oxygen consumption rate (OCR) and extracellular acidification rate (ECAR) tracing of SC under naïve and oxidative stress following treatment with capsaicin. (**E**) Mitochondrial respiratory parameter quantified as ATP production rate and spare respiratory capacity following treatment with capsaicin under naïve and oxidative stress. (**F**) Quantification of extracellular acidification rate showing non-glycolytic acidification and glycolysis. Statistical significance was assessed by the Mann–Whitney test. Data is provided as mean ± SD.

When looking at changes in mRNA expression, treatment of SC with capsaicin for 8 h resulted in a significant increase in expression of *Nrf2* (*p* < 0.0001, *n* = 12, Figure 3A), *HO-1* (*p* < 0.0001, *n* = 11, Figure 3A), and *Nqo1* (*p* < 0.0001, *n* = 11, Figure 3A), while catalase (*Cat*) expression remained unchanged. In contrast, prolonged exposure to capsaicin for 24 h led to downregulation of *Nrf2* (*p* = 0.006, *n* = 9, Figure 3B), *Ho-1* (*p* = 0.0098, *n* = 9, Figure 3B), and catalase (*Cat*) (*p* = 0.0051, *n* = 9, Figure 3B), but not to *Nqo1* expression. Notably, under oxidative stress, capsaicin treatment did not influence the expression of these antioxidative markers (Figure 3C). For an in-depth examination of capsaicin’s effect on SC, we analyzed the influence on the cells’ mitochondrial function.

Under naïve conditions, capsaicin enhanced Schwann cell bioenergetics, as evidenced by an observed increase in ATP production (*p* = 0.0117, *n* = 19, Figure 3E) and spare respiratory capacity (*p* = 0.0003, *n* = 19, Figure 3E). The same tendencies were also seen under SNAP-induced oxidative stress conditions without reaching significant levels (Figure 3E). The observed changes in ATP production are mediated via a glycolysis-independent process since non-glycolytic extracellular acidification is significantly increased upon capsaicin treatment (*p* = 0.0032, *n* = 19, Figure 3F), but the glycolysis rate remained unaffected.

### 3.4. Capsaicin Differentially Influences Schwann Cell Phenotypes in Response to Oxidative Stress

**Figure 4:**
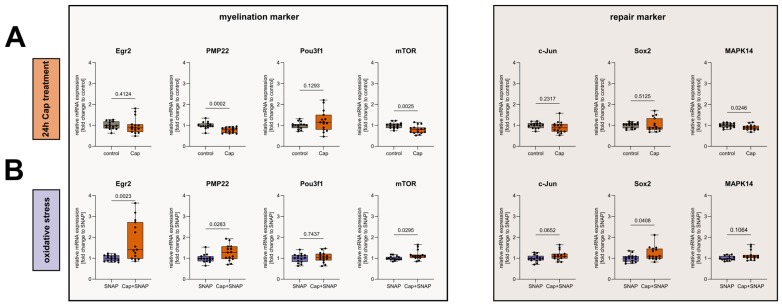
Capsaicin differentially modulates Schwann cell myelination and repair markers under naïve and oxidative-stress conditions. (**A**) Relative mRNA expression of myelination and repair markers following capsaicin treatment for 24 h under naïve conditions. (**B**) Relative mRNA expression of myelination and repair markers following capsaicin treatment under oxidative stress. Statistical significance was assessed by the Mann–Whitney test. Data are provided as mean ± SD.

To assess the phenotypic status of SC, cells exposed to capsaicin for 24 h under naïve and SNAP conditions were analyzed for changes in mRNA expression. Under naïve conditions, capsaicin-treated SC showed a decreased expression of the myelination-associated markers Peripheral Myelin Protein 22 (*Pmp22*) (*p* = 0.0002, *n* = 15, Figure 4A) and mechanistic Target of Rapamycin (*mTOR*) (*p* = 0.0025, *n* = 15, Figure 4A), along with decreased levels of mitogen-activated protein kinase 14 (*Mapk14*) (*p* = 0.0246, *n* = 15, Figure 4A).

Under oxidative stress, capsaicin treatment induced an upregulation of myelination markers Early Growth Response 2 (*Egr2*) (*p* = 0.0023, *n* = 15, Figure 4B), *Pmp22* (*p* = 0.0283, *n* = 15, Figure 4B), and *mTOR* (*p* = 0.0295, *n* = 15, Figure 4B), as well as an increase in the expression of characteristic repair-phenotype transcription factors *c-Jun* (*p* = 0.0652, *n* = 15, Figure 4B) and SRY-box transcription factor 2 (*Sox2*) (*p* = 0.0408, *n* = 15, Figure 4B).

## 4. Discussion

In our current study, we demonstrated a neuroprotective and regenerative role of the TRPV1 agonist capsaicin on DRG in vitro. Capsaicin conferred a context-specific antioxidative effect on DRG and initiated an antioxidative repair function on SC, improving the restoration of neuronal integrity.

In DRG, capsaicin mediated these antioxidative effects by upregulating catalase and activating the *Nrf2* pathway along with its downstream effectors *Ho-1* and *Nqo1*. Furthermore, capsaicin treatment induced neuroregeneration upon oxidative stress via upregulation of *Gap43* and *Atf3*.

These results reveal a major multifaceted role for capsaicin as a neuroprotective and regenerative substance for damaged nerves in the PNS and as a protective factor against oxidative stress. The findings further extend and complement our knowledge of the role of capsaicin regarding the immunomodulatory effects of capsaicin in vivo through oral application in EAN, as well as its immunomodulatory and catalase-mediated antioxidative effects on SC [21,22].

The induction of cytoprotective markers on naïve neurons and under oxidative stress through the action of capsaicin provides the conditions to protect neurons, as well as restore integrity when neuronal structure and function are impaired. Capsaicin provides a sustained expression of cytoprotective *Nrf2*, *Ho-1*, and *Nqo1* in naïve DRG, thereby creating a favorable internal milieu to withstand neuronal injury.

The transcription factor NRF2 mediates neuronal and glial cell stress adaptation by activating the expression of genes involved in antioxidant defense like *Ho-1* and *Nqo1*, enhancing cellular metabolism by modulating NADH levels and cellular detoxification [32]. Nrf2-inducers have been shown to counteract oxidative stress in several models of neurodegenerative diseases both in vitro and in vivo [33,34,35]. Additionally, capsaicin has been established to activate NRF2/antioxidant response elements in non-neuronal cells, increase translocation of NRF2 into the nucleus, inhibit the degradation of Nrf2, and disrupt the interaction between NRF2 and its inhibitor KEAP1 [36].

The sustained effect of capsaicin on *Nrf2/Ho-1/Nqo1* in DRG is mediated by the temporal transcriptional regulation of *Trpv1* in DRG, with increased expression in the ‘acute state’—8 h—and downregulation in the ‘chronic state’—24 h—of stimulation. This temporal pattern suggests early TRPV1 sensitization followed by a homeostatic downregulation of channel expression to prevent neuronal calcium overload, while still leaving the antioxidative effect active. Clinically, this mechanism has been employed in the management of pain while restoring neuronal integrity in the late term. High-dose capsaicin, which has a short elimination half-life, induces “defunctionalization,” a process that includes several sequential effects, such as desensitization of the capsaicin receptor TRPV1, transient loss of membrane potential, impaired neurotrophic factor transport leading to phenotypic changes, and reversible retraction of epidermal and dermal nerve fiber terminals. However, after three months, small skin fiber density increases markedly following the initial tissue damage [23]. In the EAN model, TRPV1 immunostaining is upregulated in the sciatic nerve at the peak of the disease, while *Trpv1* mRNA levels are concomitantly downregulated, suggesting an intrinsic compensatory transcriptional response [27].

EAN animals treated with capsaicin during the peak of the disease have an increased *Trpv1* mRNA expression, suppressed expression of pro-inflammatory cytokines (*Tnf-α*, *Ifng*), and enhanced anti-inflammatory cytokine (*Il4*) expression, with ultimately improved clinical disease severity [21]. These observations support the notion that capsaicin induces sustained *Trpv1* expression under conditions of oxidative stress, contributing to immunomodulation and neuroprotection. Therefore, the natural enhancement of TRPV1 in the context of EAN and in the context of capsaicin treatment implies its role as a neuroprotective, anti-inflammatory, and regenerative modulator.

Importantly, oxidative stress induction with SNAP following capsaicin pretreatment altered antioxidant response from the Nrf2-pathway observed under naïve conditions toward a modest increase in catalase (*Cat*) and *Trpv1* expression with improved functional neurite outgrowth. SNAP is a nitric oxide donor known to induce nitrosative stress with the accumulation of reactive nitrogen species, mitochondrial reactive oxygen species, and hydrogen peroxide (H_2_O_2_) [37]. Catalase is a core antioxidant enzyme that catalyzes the decomposition of hydrogen peroxide in cells, and its preferential upregulation may reflect a more direct response to acute peroxide burden compared with the phase II detoxification response mediated by *Nrf2* under naïve conditions [38,39,40,41]. Because the mRNA changes in catalase and *Trpv1* in this context were modest and did not exceed a two-fold threshold, this is interpreted as a transcriptional response rather than a protein-level mechanism. Moreover, catalase plays a crucial antioxidant defense role in autoimmune diseases and age-associated degenerative diseases [8,38]. In naïve Schwann cells and DRG exposed to propionate, a short-chain fatty acid, catalase expression was significantly increased, with no changes in the expression of *Ho-1* and *Nqo1*. Furthermore, propionate pre-treatment enhanced neurite outgrowth in DRG neurons subjected to SNAP-induced damage [8]. Collectively, these findings suggest that catalase is selectively modulated by nutritive factors to confer neuronal protection. The sustained upregulation of *Trpv1* mRNA in DRG neurons observed at 24 h under oxidative stress may be driven by the activation of oxidative stress-derived products [42,43], in contrast to naïve conditions, where prolonged exposure to capsaicin leads to downregulation of *Trpv1* at 24 h.

Treatment with capsazepine, a competitive TRPV1 antagonist, partially abolished the enhanced axonal growth of capsaicin on DRG neurons under oxidative stress, thereby indicating a TRPV1-mediated mechanism. However, given that not all DRG neuronal subtypes express TRPV1 and given the heterogeneity in the expression of TRPV1 in distinct TRPV1-positive neuron subpopulations in DRG [44], the specific neuronal subtypes contributing to this phenotype remain unclear. Future studies incorporating specific neuronal subpopulations with distinct neurochemical and morphological features could help to further elucidate the underlying mechanisms in TRPV1-mediated neuroprotection.

We demonstrated a restoration of axonal growth after SNAP treatment, as well as increased expression of *Gap43* and *Atf3*, both markers for regeneration and plasticity in neurons. Examining the effects of capsaicin treatment in naïve DRG neurons, an early increase in *Gap43* expression after 8 h, followed by upregulation of *Atf3* after 24 h, indicates a temporally coordinated activation of the neuronal regenerative program before oxidative stress exposure. Consistent with previous findings, Frey et al. reported that the concentration of 10 µM capsaicin improved axonal outgrowth and induced pro-regenerative marker *Scg10* in dissociated mouse DRG neurons [45]. In our explant model, treatment with 10µM capsaicin did not significantly increase axonal outgrowth; however, it robustly upregulated regeneration-associated markers *Gap43* and *Atf3*. This dissociation between morphological and molecular outcomes suggests that capsaicin may prime DRG neurons toward a pro-regenerative state without being sufficient to drive axonal extension in DRG explant cultures. This early pro-regenerative transcriptional response is consistent with an observed reduction in disease severity in EAN following early capsaicin dietary preventive therapy [21]. The present findings, which suggest that capsaicin may have neuroprotective, antioxidative, and regenerative properties, could be relevant in the management of immune-mediated neuropathies like CIDP. However, given that the present study was conducted exclusively in vitro, this translational interpretation remains speculative. Though epidemiological studies have reported regional differences in immune-mediated neuropathies like Guillain-Barré Syndrome (GBS) in relation to capsaicin consumption, with higher incidence in Europe and North America in comparison to China and Brazil, such associations are indirect and do not establish causality. Further in vivo and clinical studies are required to determine whether the cellular effects observed with capsaicin in vitro have therapeutic and preventive relevance [13,14,15].

Complementary to our investigation of DRG, we characterized SC in response to treatment with capsaicin. We demonstrated the bioenergetic profile of mitochondrial function within naïve SC under the influence of capsaicin, marked by increased ATP production and increased spare respiratory capacity. The enhanced respiratory reserve observed characterizes the extra potential in energy production of Schwann cells that would be needed to support the high energy demands of neurons for myelin maintenance, trophic support, and other functions [46,47,48]. Such metabolic adaptations are also known to contribute to neurite outgrowth in neuroprotection and support for regenerating axons [49]. Further analysis of mitochondria in Schwann cells revealed that the increased levels of energy production by capsaicin are mediated via oxidative phosphorylation, since rates of glycolysis remained unchanged with simultaneous increases in non-glycolytic acidification measured during the assay. Extracellular acidification measured during the mitochondrial assay relies on the export of protons into the surrounding cell culture medium originating from ATP production in the cell. However, because the current study is limited to Schwann cell monocultures and does not include DRG/SC cocultures or functional axonal-support assays, the direct benefits of these SC bioenergetics changes for neuroprotection and neuroregeneration cannot be definitively concluded. This should be interpreted as evidence of changes in SC metabolic state induced by capsaicin. Future studies of DRG/SC cultures or functional assays will help determine whether these SC metabolic changes from capsaicin directly impact neuroregeneration and neuroprotection.

Capsaicin not only modulated the oxidative defense system and metabolism but also shaped the developmental state of Schwann cells in a context-dependent fashion. Under naïve conditions, 24 h capsaicin treatment led to the downregulation of markers *Pmp22*, *mTOR*, and *Mapk14*. The expression of *Pmp22* in Schwann cells is mandatory for proper development and differentiation, especially in forming a functional myelin sheath. Dysregulation of PMP22 expression is known to cause severe hereditary neuropathy, Charcot–Marie–Tooth disease [50]. Both mTOR and MAPK14 have multiple roles in the physiology of Schwann cells and their presentation in different phenotypes. Constant mTOR expression in Schwann cells is important in differentiation processes towards myelinating Schwann cells [51]. Nevertheless, in response to injury, it has been shown that mTOR and MAPK14 also function as differentiation markers towards the induction of “repair-like” Schwann cells in several pathway interactions [52]. Taken together, the conservation in marker expression of *Pmp22*, *mTOR*, and *Mapk14* neither indicates a myelination nor a repair phenotype induced by capsaicin in Schwann cells under naïve conditions. Non-myelinating Schwann cells, also known as Remak Schwann cells, serve as homeostatic support for thin, unmyelinated C fibers in the nervous system. Since these fibers are well characterized in their TRPV1 expression [53], this implies that, via potential intercellular crosstalk between neurons and Schwann cells, capsaicin can help maintain Schwann cells in the Remak state near neurons with high TRPV1 expression.

Under oxidative stress, significant upregulation of *mTOR*, *Egr2*, *Pmp22*, and *Sox2*—together with trends toward increased *c-Jun* and *Mapk14* levels—was observed. The simultaneous upregulation of myelination-inducing factors *Pmp22*, *mTOR*, and *Egr2* with typical repair-Schwann cell markers *c-Jun* and *Sox2* represents a complex interaction of different, opposing pathways in Schwann cells. Since our results only consider the mRNA expression at one timepoint without longitudinal comparison of capsaicin treatment, certain functional indications may be temporally overlapping. After injury, repair-associated markers *c-Jun* and *Sox2* are upregulated in Schwann cells to mediate the regeneration of peripheral nerves [54]. Subsequently, myelin-related genes are upregulated to facilitate remyelination, especially characterized by *Egr2* and downstream targets [55]. Taken together, our results present capsaicin as an agent that sustains the homeostatic functions of Schwann cells under naïve conditions and induces de-differentiation upon oxidative stress, with increased metabolic function resulting in increased ATP production.

These desirable effects of capsaicin on SCs are highly relevant as SCs lose their plasticity in immune-mediated neuropathies, with impaired pro-repair and supportive functions leading to incomplete regeneration of injured axons [56]. Therefore, capsaicin treatment may be beneficial in enhancing the plasticity of Schwann cells in degenerative and inflammatory neuropathies to promote recovery. Although protein level analyses of TRPV1 and downstream markers would strengthen the mechanistic interpretation of our findings, these observations are supported by functional neurite outgrowth assay changes that correlate with mRNA changes in anti-oxidant, regenerative-related genes in DRG neurons and myelination and repair-related genes in Schwann cells, providing consistent evidence of capsaicin-mediated neuroprotection, regeneration in DRG, and improved SC bioenergetics.

Overall, our findings support a framework in which capsaicin—via TRPV—drives resistance to oxidative stress and a regenerative program in DRG neurons, with a glial compartment (SC) that provides complementary support for a phenotype matching the local environment and neuronal needs. These observations are of extreme importance for a further group of polyneuropathies, which involve small nerve fibers (C- and A-delta fibers with somata in the dorsal root ganglia). Fluorescence in situ hybridization (FISH) analysis of rat DRG neurons has demonstrated heterogeneous TRPV1 expression patterns across sensory neuron subtypes, with small fibers like C fibers exhibiting higher expression of *Trpv1* mRNA than large diameter fibers [44]. Based on our results, *Trpv1* expression is upregulated during oxidative stress to counteract the danger of degeneration and improve neuroprotection. Capsaicin treatment may favor this pathway and play a major preventive or therapeutic role.

## 5. Conclusions

In the final analysis, we have shown that capsaicin exerts antioxidative, neuroprotective, and regenerative effects on DRG and enhances SC metabolic profile and plasticity. Further studies must determine whether changes in nutrition in favor of spicy food are able to exert a protective and regenerative effect on human polyneuropathies. Moreover, it may be possible that capsaicin modulates the disease course of patients with immune-mediated polyneuropathy after long-term nutritional changes. To clarify the entire effect of capsaicin on inflammatory, autoimmune-mediated, and degenerative neuropathies, further studies should examine alternative cellular pathways following TRPV1 activation in the context of the different stages of inflammation and degeneration for different cell types in PNS.

## Figures and Tables

**Table 1:** List of primers for all gene targets.

Gene	Sense Primer	Antisense Primer
*Gapdh*	AGG TCA CCC AGA GCT GAA CG	CAC CCT GTT GCT GTA GCC GTA T
*Actb*	CCC ATC TAT GAG GGT TAC GC	TTT AAT GTC ACG CAC GAT TTC
*Trpv1*	CTT CTG AGG GAT GCA AGC AC	CCT GGG ACC ATG GAA TCC TT
*Nrf2*	CTC TCT GGA GAC GGC CAT	CTG GGC TGG GGA CAG TGG
*Cat*	CGG CAC ATG AAT GGC TAT GG	TGC CCT GGT CAG TCT TGT AAT
*Ho-1*	AGG AAA ATC CCA GAT CAG CAG	GAA AAG AGA GCC AGG CAA GAT
*Nqo1*	GTT TCT TTT TCC CCA GTT TGC	GGC TAC ACC TCT CCC TGA TTC
*Gap43*	CTC TCC TGC CCT TTC TCA GAT	ACT CGC CAT AAC AAC AAC AAG
*Atf3*	TGT ACC CAC TGC TGA GGA AG	GTC CTA CGC CCT TTG AAA GC
*c-Jun*	ACT GGT TGC GAC AGA GAA AAA	CGA ATG TTA GGT CCA TGC AGT
*Egr2*	CCT TCC CTT TGA CCC TCG AT	AGT CCG TCC CAA GCC ATT AA
*Pmp22*	TCT CAA AGC CTT CGT CAC TCC	GTG GCC AAT ACA AGT CAT CGC
*Pou3f1*	TCG TTT CGT TTT ACC AGA GCC	CTC GAT CTT GCG GGT GAA G
*mTor*	TGT GTT CAG TGA GGG ATG GA	ATT CAC AAC CTG CGC TAG TG
*Sox2*	TAA TCA CAA CAA TCG CGG CG	CTG GCG GAG AAT AGT TGG GG
*Mapk14*	CAG TAC CAC GAC CCT GAT GA	ATC GTA GGT CAG GCT CTT CC

## Data Availability

The original contributions presented in this study are included in the article/Supplementary Materials. Further inquiries can be directed to the corresponding author(s).

## Supplementary Materials

The following supporting information can be downloaded at: https://www.mdpi.com/article/10.3390/cells15131142/s1, Figure S1: Titration analysis of different concentrations of capsaicin; Figure S2: Cross-sectional immunohistochemical staining of naïve rat DRG.
