# Supplementary figures and images for: TRPV1 Agonist Capsaicin Enhances Oxidative-Stress Resistance and Regeneration in Dorsal Root Ganglia and Schwann Cells
Source: Cells. 2026 Jun 24;15(13):1142. doi: 10.3390/cells15131142 (PMC13360480; doi:10.3390/cells15131142)

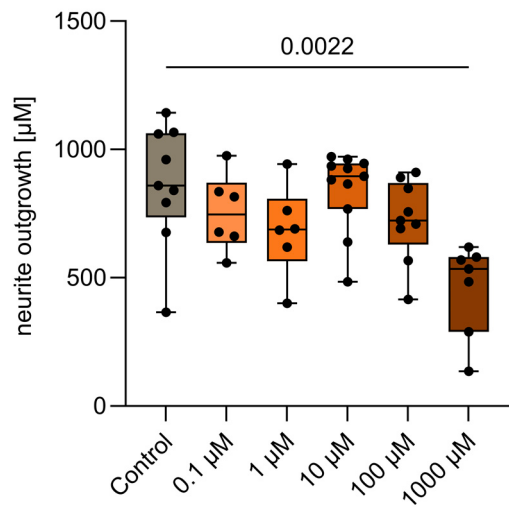

### DRG

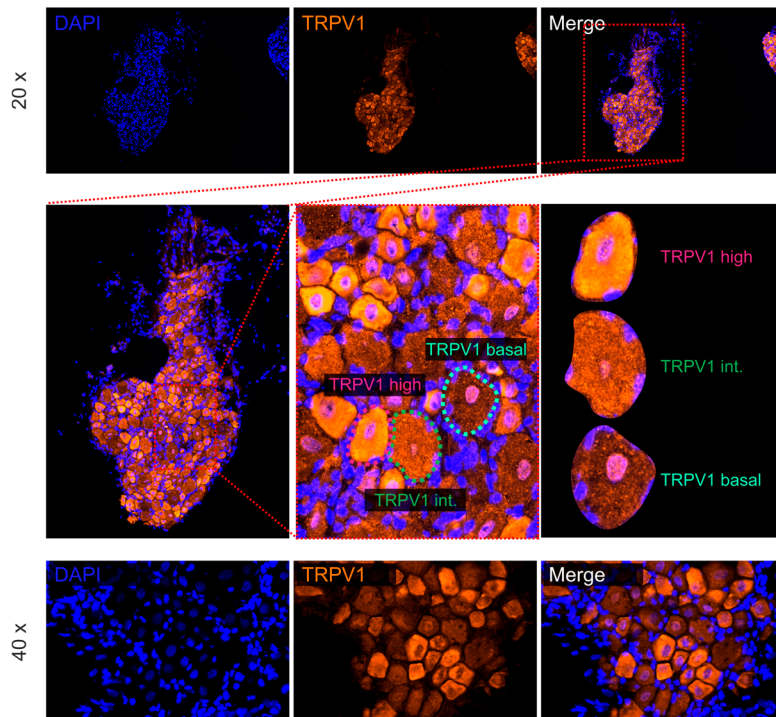

Supplement: Supplementary file 1 [file cells-15-01142-s001.zip › cells-4312583-supplementary.pdf]
